# Levo-tetrahydropalmatine attenuates oxaliplatin-induced mechanical hyperalgesia in mice

**DOI:** 10.1038/srep03905

**Published:** 2014-01-28

**Authors:** Zhenggang Guo, Yuanyuan Man, Xiaoyan Wang, Heng Jin, Xuefeng Sun, Xiaojun Su, Jianhua Hao, Weidong Mi

**Affiliations:** 1 Department of Anesthesiology, The First Hospital Affiliated to the Chinese PLA Hospital, Beijing, 100048, China; 2 Anesthesia and Operation Center, Chinese PLA General Hospital, Beijing, 100853, China; 3 These authors contributed equally to this work.

## Abstract

Common chemotherapeutic agents such as oxaliplatin often cause neuropathic pain during cancer treatment in patients. Such neuropathic pain is difficult to treat and responds poorly to common analgesics, which represents a challenging clinical issue. *Corydalis yanhusuo* is an old traditional Chinese medicine with demonstrated analgesic efficacy in humans. However, the potential analgesic effect of its active component, levo-tetrahydropalmatine (*l*-THP), has not been reported in conditions of neuropathic pain. This study found that *l*-THP (1–4 mg/kg, i.p.) produced a dose-dependent anti-hyperalgesic effect in a mouse model of chemotherapeutic agent oxaliplatin-induced neuropathic pain. In addition, we found that the anti-hyperalgesic effect of *l*-THP was significantly blocked by a dopamine D_1_ receptor antagonist SCH23390 (0.02 mg/kg), suggesting a dopamine D_1_ receptor mechanism. In contrast, *l*-THP did not significantly alter the general locomotor activity in mice at the dose that produced significant anti-hyperalgesic action. In summary, this study reported that *l*-THP possesses robust analgesic efficacy in mice with neuropathic pain and may be a useful analgesic in the management of neuropathic pain.

Chemotherapy for cancer treatment often induces a form of unique peripheral neuropathy characterized by provoked and ongoing pain, which is increasingly considered as a serious side effect associated with some chemotherapeutic agents, including taxanes, platinum agents (e.g., oxaliplatin) and vinca alkaloids. The occurrence rate of chemotherapy-induced neuropathic pain varies substantially from 30–75% in the cancer patients, depending on the treatment regimens. Besides pain, other common peripheral sensory symptoms include paresthesias and dysesthesias, numbness and tingling, and sensitivity to touch and temperature. In addition, motor symptoms are also reported, including weakness and gait and balance disturbances^1^. Unfortunately, this kind of neuropathic pain is only partially reversible even long after the cessation of treatment and in some rare cases damage can be permanent. Currently, there is no effective pharmacotherapies that is considered safe and widely useful for the clinical control of chemotherapy-induced neuropathic pain. Thus, the development of alternative effective analgesics has been considered a crucial clinical need.

*Corydalis yanhusuo* is a perennial herb in the Papaveraceae family and has long been used in traditional Chinese medicine. Contemporary phytochemistry studies of *Corydalis yanhusuo* started in 1960s and Hsu and Kin were the first to isolate *l*-tetrahydropalmatine (*l*-THP) from *Corydalis yanhusuo* and did the first pharmacological characterization of the compound^2^,^3^. *l*-THP has been identified as one of the major active components of *Corydalis yanhusuo* and it has been used clinically in China for more than 40 years as an analgesic with sedative/hypnotic properties^4^. However, although *l*-THP has been used for the treatment of headache and other mild pain in China, relatively few preclinical studies support these use and there is no study to examine the potential effectiveness of *l*-THP for the treatment of chronic neuropathic pain. Given the long clinical use of *l*-THP which has proved its human safety, exploring novel applications of this compound may expand its clinical usage.

In this study, we described the potent anti-hyperalgesic effect of *l*-THP in a mice model of oxaliplatin-induced neuropathic pain. We also conducted antagonist studies to understand the receptor mechanisms of the anti-hyperalgesic actions. Our results revealed a primary dopamine D_1_ receptor mediated effect.

## Results

**Figure 1:**
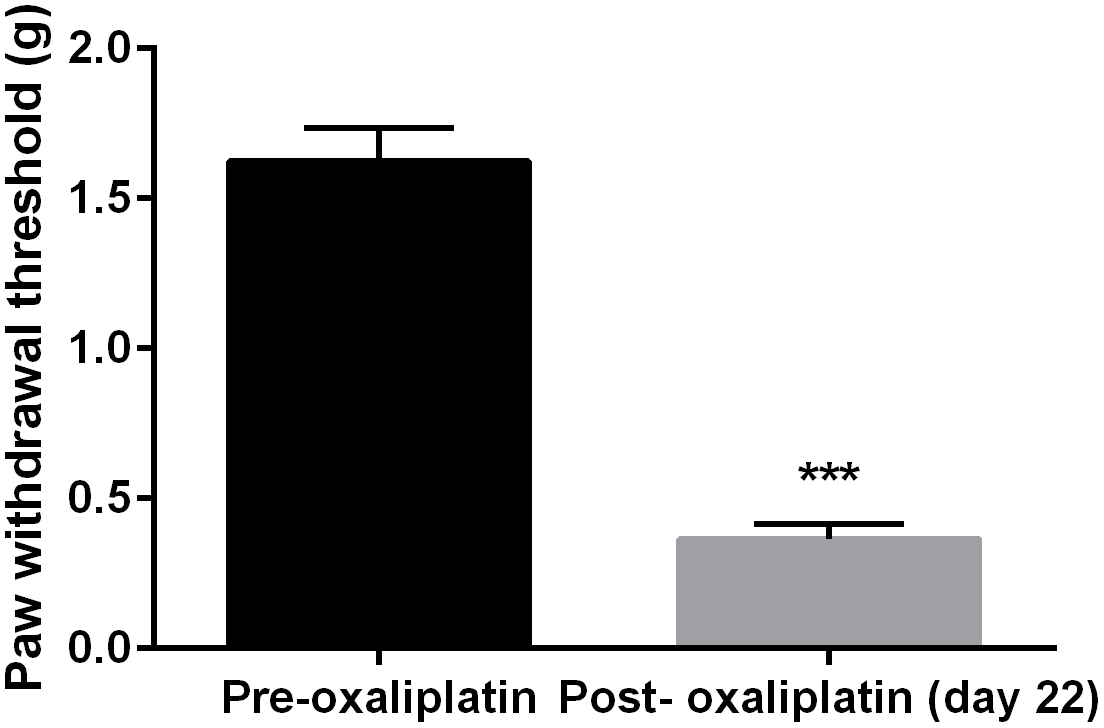
Paw withdrawal thresholds before and after oxaliplatin treatment in mice (n = 8 per group). *** P < 0.001 as compared to pre-oxaliplatin measurements.

**Figure 2:**
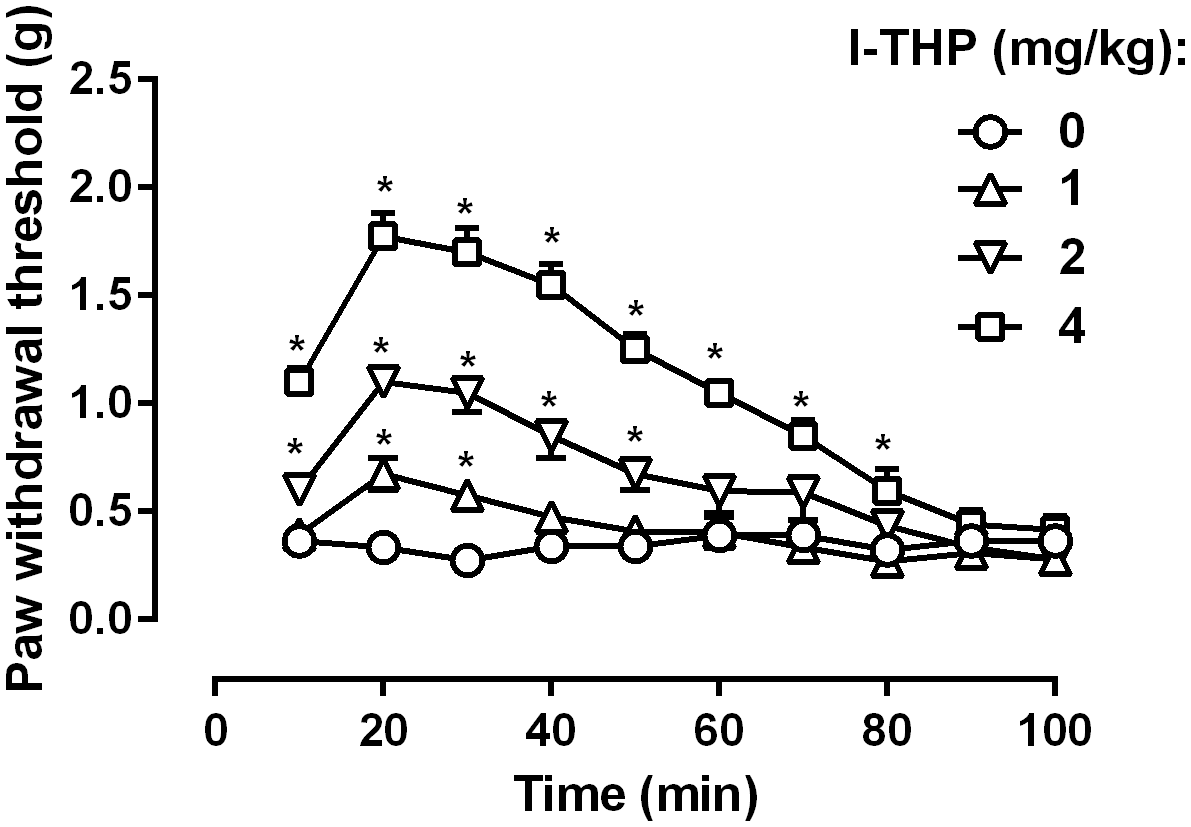
Anti-hyperalgesic effect of *l*-THP in mice (n = 8 per group). * P < 0.05 as compared to corresponding control data.

Chronic oxaliplatin treatment (3 mg/kg) led to marked mechanical hyperalgesia in mice as measured by von Frey filament (Fig. 1). Paired t-test revealed that oxaliplatin treatment produced a significant decrease in the paw withdrawal threshold (t (7) = 13.99, P < 0.0001). In addition, repeated test every 10 min over a period of 100 min did not alter the hyperalgesic condition, which remained significantly lower than the baseline measurement prior to oxaliplatin treatment (Fig. 2). Two-way ANOVA revealed significant main effects of oxaliplatin treatment (F [3, 21] = 102.3, P < 0.0001) and time (F [9, 63] = 44.44, P < 0.0001). *l*-THP dose-dependently increased the paw withdrawal threshold in mice (Fig. 2). A smaller dose of *l*-THP (1 mg/kg) only slightly elevated the paw withdrawal threshold and *post hoc* multiple analysis showed that at a dose of 1 mg/kg *l*-THP significantly increased the paw withdrawal threshold during the time points of 20 and 30 min. A larger dose of *l*-THP (2 mg/kg) markedly and significantly increased the paw withdrawal threshold. Two-way ANOVA revealed a significant main effect of *l*-THP treatment (F [1, 63] = 27.36, P < 0.0001). Multiple comparison analysis found that the paw withdrawal threshold was significantly increased throughout the 10–50 min time period. When the dose of *l*-THP was further increased to 4 mg/kg, the paw withdrawal threshold was significantly increased to the pre-oxaliplatin treatment level (Fig. 2).Two-way ANOVA revealed a significant main effect of *l*-THP treatment (F [1, 63] = 76.45, P < 0.0001). Multiple comparison analysis found that the paw withdrawal threshold was significantly increased throughout the 10–80 min time period.

**Figure 3:**
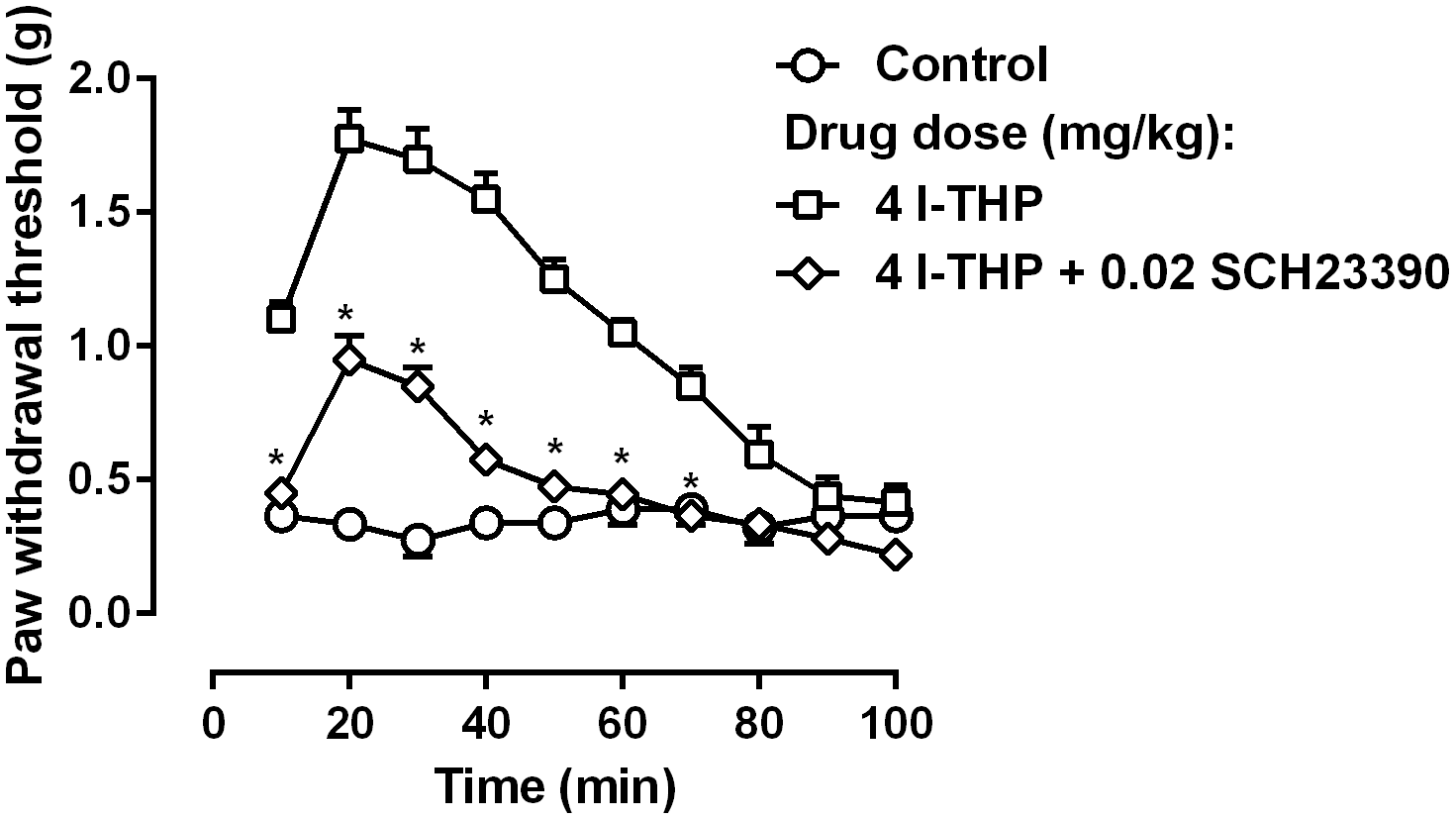
Effect of SCH23390 on 4 mg/kg *l*-THP-induced anti-hyperalgesia in mice (n = 8 per group). * P < 0.05 as compared to corresponding 4 mg/kg *l*-THP data.

In order to understand the receptor mechanism underlying the anti-hyperalgesic actions of *l*-THP, a dose of the selective dopamine D_1_ receptor antagonist SCH23390 (0.02 mg/kg) was studied in combination with 4 mg/kg *l*-THP (Fig. 3). SCH23390 significantly attenuated the anti-hyperalgesic effects of *l*-THP. Two-way ANOVA revealed that there were significant main effects of SCH23390 treatment (F [1, 14] = 158.0, P < 0.0001) and time (F [9, 126] = 60.13, P < 0.0001). Post hoc analysis found that the anti-hyperalgesic effect of *l*-THP was significantly decreased across the 10–70 min time period.

**Figure 4:**
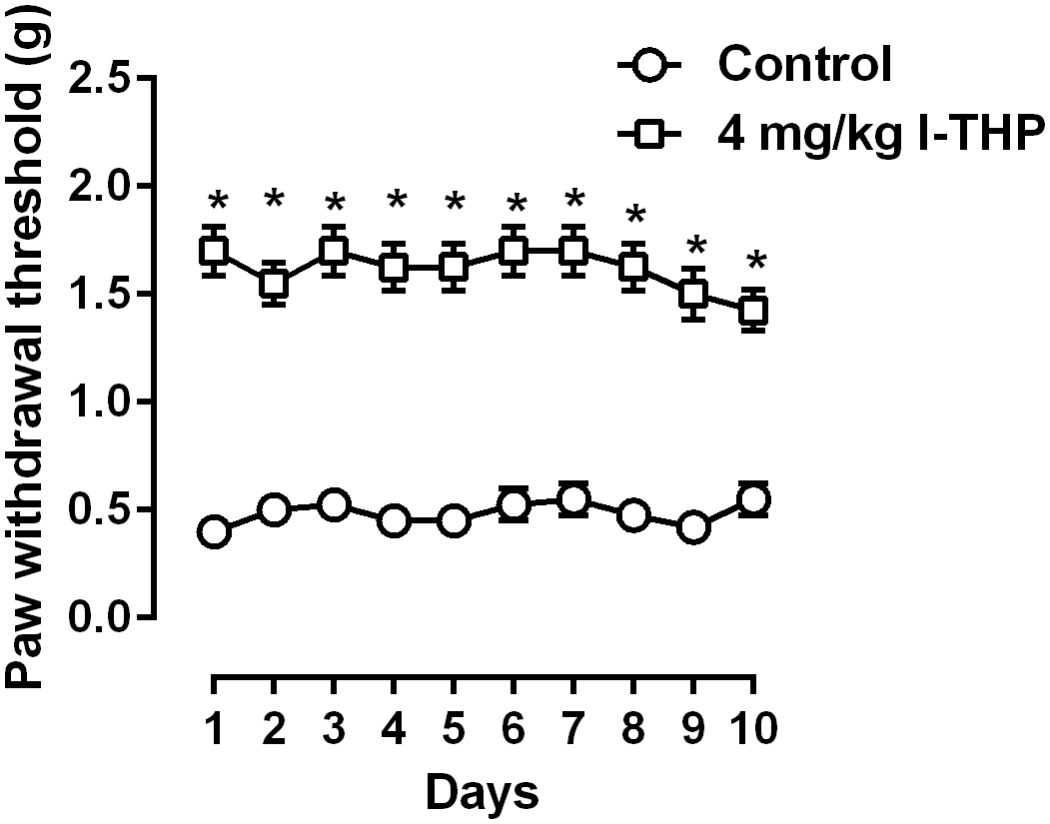
Anti-hyperalgesic effect of daily 4 mg/kg *l*-THP treatment in mice (n = 8 per group). * P < 0.05 as compared to corresponding daily baseline data as measured before *l*-THP treatment.

We also studied the anti-hyperalgesic effects of repeated daily *l*-THP treatment (Fig. 4). Daily treatment with 4 mg/kg *l*-THP, a dose that completely reversed mechanical hyperalgesia, maintained its anti-hyperalgesic effect and no significant antinociceptive tolerance was observed over a period of 10 days of daily treatment. Two-way ANOVA revealed a significant main effect of *l*-THP treatment (F [1, 7] = 103.5, P < 0.0001), but no significant main effects of time or interaction were found. Post hoc analysis found that the paw withdrawal threshold after 4 mg/kg *l*-THP treatment was significantly higher as compared to the daily pre-drug treatment baseline. In addition, the anti-hyperalgesic effect among the 10 daily treatments was not significantly different.

**Figure 5:**
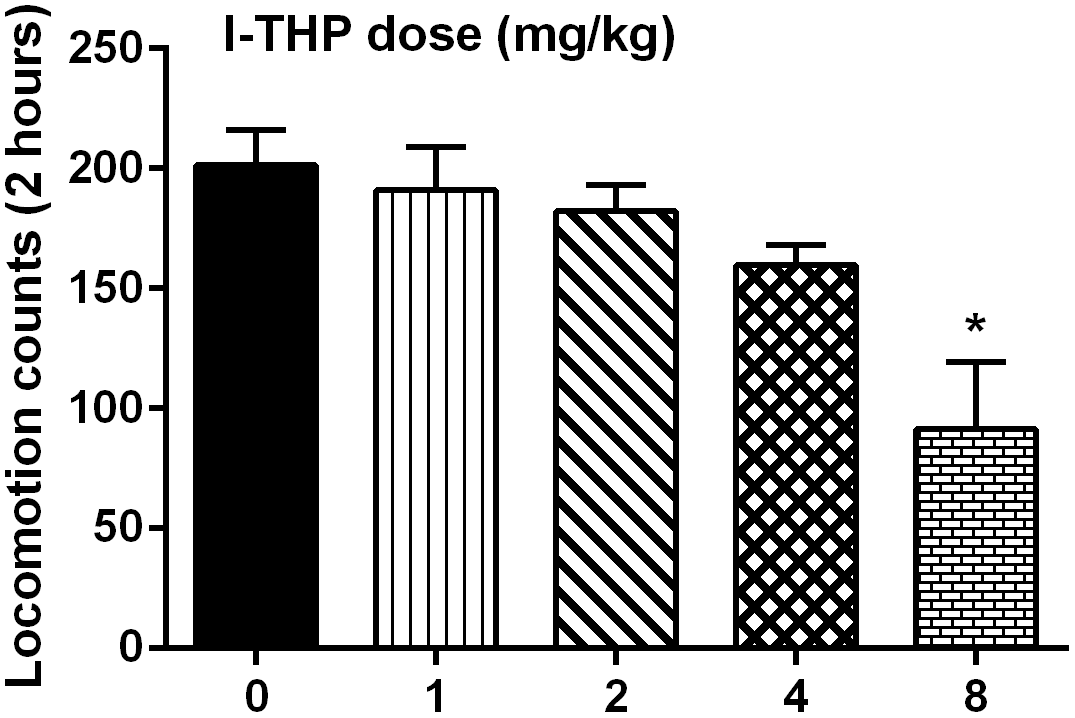
Effect of *l*-THP on general locomotor activity in mice (n = 8 per group).

The potential effect of *l*-THP treatment on the general locomotor activity in naïve mice was examined with different doses of *l*-THP (Fig. 5). It was found that *l*-THP had a dose-dependent effect in decreasing the general locomotor activity in mice. One-way ANOVA found a significant effect of *l*-THP dose (F [4, 35] = 11.48, P < 0.05). Post hoc analysis revealed that 8 mg/kg *l*-THP treatment significantly decreased the locomotor activity.

## Discussion

In this study, we reported that an active component from the plant *Corydalis yanhusuo*, *l*-THP, produced robust anti-hyperalgesic effect in a mouse model of chemotherapy-induced neuropathic pain. We also reported that the anti-hyperalgesic effect of *l*-THP was primarily mediated by dopamine D_1_ receptors and the effect was not due to general behavioral impairment. Although *l*-THP has been used in China for the treatment of mild to moderate pain including headache, this is the first study that identified the antinociceptive effects of *l*-THP in a mouse model of chemotherapeutic agent-induced neuropathic pain. In summary, although preliminary, these results support the expanding use of *l*-THP in the clinical control of neuropathic pain.

Many microtubule-targeting cancer chemotherapeutic agents including oxaliplatin has long been recognized to cause peripheral and cranial neuropathy^5^,^6^,^7^. In an effort to better understand this form of neuropathy and develop novel treatment for its management, several animal models of chemotherapeutic agent-induced neuropathy was developed^8^,^9^,^10^. Rodents treated with chemotherapeutic agents typically develop thermal and mechanical hyperalgesia. In consistency with the literature, we found that mice treated with 3 mg/kg intermittently for 10 injections developed a reliable mechanical hyperalgesia as measured by von Frey filament test. Repeated measures within a short period of time (100 min) did not significantly change the test results, which offers an opportunity to determine the duration of actions of the study drug. We found that *l*-THP produced a robust effect in decreasing mechanical hyperalgesia. This effect was both dose-dependent and time-dependent and at larger doses it completely reversed the mechanical hyperalgesia. This represents the first study that clearly demonstrated that *l*-THP has very robust antinociceptive effect in a mouse model of chronic neuropathic pain. In addition, the observed anti-hyperalgesic effects were not due to general behavioral suppression because within the dose range studied, *l*-THP did not significantly alter the locomotor activity in mice. More importantly, repeated treatment with *l*-THP did not show the development of antinociceptive tolerance. Considering the long-term therapeutic need to treat neuropathic pain, this lack of tolerance development is significant and clearly supports the use of *l*-THP in the treatment of chronic neuropathic painful conditions.

Early receptor binding studies suggest that *l*-THP binds to dopamine D_1_ and D_2_ receptors at relatively high affinity and binds to serotonergic 5-HT_1A_ receptors at lower affinity^2^. In a rat radiant tail flick test, *l*-THP produced a dopamine D_2_ receptor mediated antinociception^11^. This study found that a selective dopamine D_1_ receptor antagonist, SCH23390, significantly blocked the anti-hyperalgesic effect of *l*-THP, suggesting that the anti-hyperalgesic action of *l*-THP is primarily mediated by activating dopamine D_1_ receptors. This discrepancy may be due to the pain model used (radiant tail flick versus neuropathic pain), species (rats versus mice) and the doses studied (10–40 mg/kg versus 1–4 mg/kg).

In summary, this study for the first time demonstrated that *l*-THP has a potent antinociceptive effect in a mouse model of chemotherapeutic agent-induced neuropathic pain, without apparent adverse effects (motor impairment). Although more studies are needed to examine the generality of these findings, because *l*-THP has been used in China for over 40 years and its human safety has been clearly demonstrated, the current data suggest to explore the expansion of *l*-THP for the treatment of neuropathic pain.

## Methods

### Animals

Male C57BL/6 mice weighing 16–22 g (Weitong Lihua, Beijing, China) were acclimated to the temperature, humidity and lighting (12 h light/dark cycle, lights on at 7:00 AM) controlled vivarium and housed in groups of four for at least one week before behavioral studies began. The animals had free access to dietary food and water except during the test sessions. All animal experimental protocols were approved by the local Institutional Animal Care and Use Committee, The First Hospital Affiliated to the Chinese PLA Hospital. Animals were maintained and experiments were conducted in accordance with the *Guide for the Care and Use of Laboratory Animals* (8^th^ edition, Institute of Laboratory Animal Resources on Life Sciences, National Research Council, National Academy of Sciences, Washington DC). All efforts were made to minimize animal suffering and to reduce the number of animals used.

### Drugs

Oxaliplatin (Sigma-Aldrich, St. Louis, MO) was dissolved in 5% dextrose (1 mg/ml) and prepared fresh for daily use. Levo-tetrahydropalmatine (*l*-THP) was purchased from Shanghai Lei Yun Shang Pharmaceutical Co. (>95% purity, Shanghai, China). SCH23390 was purchased from Sigma-Aldrich (St. Louis, MO, USA) and dissolved in saline. *l*-THP was dissolved in saline with one drop of acetic acid. Except otherwise noted, all injections were given intraperitoneally in a volume of 1 ml/100 g of body weight. After habituation to the test environment and baseline measurements of pain sensitivity, mice were randomized to two treatment conditions of either oxaliplatin (3.0 mg/kg) or vehicle (0.9% saline). Using injection volume of 10 ml/kg, mice were treated with daily administration for 5 days, followed by 5 days of rest, for two weekly cycles. Total cumulative dose of 30 mg/kg oxaliplatin over a total of ten injections was used.

### Mechanical hyperalgesia measurement

Mechanical hyperalgesia was assessed prior to and 1 day after the last oxaliplatin treatment using Von Frey filaments of varying forces (0.07–4.0 g) applied to the mid-plantar surface of the right hind paw, with each application held until curved for 6 s using the up-down method^2^. Mice were placed in individual Plexiglas compartments atop of a wire grid floor suspended 50 cm above the laboratory bench top and acclimated to the environment for 30 min prior to each test session. For the time course studies, baseline von Frey filament measurement was immediately followed by an injection of *l*-THP, and then the paw withdrawal threshold was measured every 10 min until the drug effect dissipated to a point that the paw withdrawal threshold was not significantly different from the pre-drug data. In studies that test the effect of the antagonist SCH23390, drug was administered 10 min prior to *l*-THP treatment and a time course measurement was followed. For repeated treatment studies, mice were measured daily before drug treatment and 30 min after drug treatment for 10 days.

### Locomotor activity test

The locomotor activity of naïve mice treated with vehicle or *l*-THP was measured automatically with a Small Animal Locomotion Recording Apparatus (Shandong Academy of Medical Sciences, China), which consisted of six acrylic boxes and in each box there was one pyroelectric infrared sensor 4 cm above the floor. The sensor could detect the movements of the mice through infrared radiation. The apparatus recorded only gross movements of the mice, whereas small movements such as gnawing or grooming could not be differentiated and recorded.

### Data analyses

For the mechanical hyperalgesia test prior to and 1 day after the last oxaliplatin treatment, data were analyzed using paired t-test. For the antinociceptive studies, data were presented as paw withdrawal threshold (grams) plotted as a function of time (min or days), respectively. Data were analyzed by two-way repeated measures analysis of variance (ANOVA) (time × *l*-THP treatment or time × oxaliplatin treatment) followed by post hoc Bonferroni test. For the locomotion tests, data were analyzed with one-way ANOVA followed by post hoc Bonferroni test.

## Author Contributions

Z.G., J.H. and W.M. designed the experiments; Z.G., Y.M., X.W., H.J. and X.S. conducted the experiments; Z.G., X.S., J.H. and W.M. wrote the main manuscript text; Y.M. and X.W. conducted the statistical analyses and prepared the figures. All authors reviewed and approved the manuscript.

## References

[b1] Visovsky C., Collins M., Abbott L., Aschenbrenner J. & Hart C. Putting evidence into practice: evidence-based interventions for chemotherapy-induced peripheral neuropathy. Clin. J. Oncol. Nurs. 11, 901–913 (2007). 18063548 10.1188/07.CJON.901-913

[b2] Hsu B. & Kin K. C. Pharmacological study of tetrahydropalmatine and its analogs. A new type of central depressants. Arch. Int. Pharmacodyn. Ther. 139, 318–327 (1962). 13955299

[b3] Hsu B. & Kin K. C. Some pharmacological properties of corydalis B (tetrahydropalmatine) and its related compounds. Sci. Sin. 13, 601–609 (1964). 14168947

[b4] Jin G. Z. *l*-Tetrahydropalmatine and its analogues as new dopamine receptor antagonists. Trends Pharmacol. Sci. 8, 81–82 (1987).

[b5] Dixit G., Dhingra A. & Kaushal D. Vincristine induced cranial neuropathy. J. Assoc. Physicians India 60, 56–58 (2012). 22799120

[b6] Carlson K. & Ocean A. J. Peripheral neuropathy with microtubule-targeting agents: occurrence and management approach. Clin. Breast Cancer 11, 73–81 (2011). 21569993 10.1016/j.clbc.2011.03.006

[b7] Jaggi A. S. & Singh N. Mechanisms in cancer-chemotherapeutic drugs-induced peripheral neuropathy. Toxicology 291, 1–9 (2012). 22079234 10.1016/j.tox.2011.10.019

[b8] Jaggi A. S. & Singh N. Analgesic potential of intrathecal farnesyl thiosalicylic acid and GW 5074 in vincristine-induced neuropathic pain in rats. Food Chem. Toxicol. 50, 1295–1301 (2012). 22326968 10.1016/j.fct.2012.01.038

[b9] Nativi C. *et al.* A TRPA1 antagonist reverts oxaliplatin-induced neuropathic pain. Sci. Rep. 3, 2005 (2013). 23774285 10.1038/srep02005 PMC3684817

[b10] Contreras P. C. *et al.* Insulin-like growth factor-I prevents development of a vincristine neuropathy in mice. Brain Res. 774, 20–26 (1997). 9452187 10.1016/s0006-8993(97)81682-4

[b11] Hu J. Y. & Jin G. Z. Supraspinal D2 receptor involved in antinociception induced by *l*-tetrahydropalmatine. Zhong Guo Yao Li Xue Bao 20, 715–719 (1999). 10678104

